# Microbiome profile associated with malignant pleural effusion

**DOI:** 10.1371/journal.pone.0232181

**Published:** 2020-05-08

**Authors:** Samira Shojaee, Anukriti Sharma, Neil Gottel, Trinidad Sanchez, Jack A. Gilbert, Najib M. Rahman

**Affiliations:** 1 Division of Pulmonary and Critical Care Medicine, Virginia Commonwealth University Medical Center, Richmond, VA, United States of America; 2 Department of Pediatrics, University of California San Diego School of Medicine, San Diego, CA, United States of America; 3 Scripps Institution of Oceanography, UCSD, San Diego, CA, United States of America; 4 Oxford Respiratory Trials Unit, Oxford Centre for Respiratory Medicine, University of Oxford, Oxford, United Kingdom; 5 Oxford NIHR Biomedical Research Centre, Oxford, United Kingdom; Medical University of Graz, AUSTRIA

## Abstract

**Introduction:**

There is ongoing research into the development of novel molecular markers that may complement fluid cytology malignant pleural effusion (MPE) diagnosis. In this exploratory pilot study, we hypothesized that there are distinct differences in the pleural fluid microbiome profile of malignant and non-malignant pleural diseases.

**Method:**

From a prospectively enrolled pleural fluid biorepository, samples of MPE were included. Non-MPE effusion were included as comparators. 16S rRNA gene V4 region amplicon sequencing was performed. Exact Sequence Variants (ESVs) were used for diversity analyses. The Shannon and Richness indices of alpha diversity and UniFrac beta diversity measures were tested for significance using permutational multivariate analysis of variance. Analyses of Composition of Microbiome was used to identify differentially abundant bacterial ESVs between the groups controlled for multiple hypothesis testing.

**Results:**

38 patients with MPE and 9 with non-MPE were included. A subgroup of patients with metastatic adenocarcinoma histology were identified among MPE group (adenocarcinoma of lung origin (LA-MPE) = 11, breast origin (BA-MPE) = 11). MPE presented with significantly greater alpha diversity compared to non-MPE group. Within the MPE group, BA-MPE was more diverse compared to LA-MPE group. In multivariable analysis, ESVs belonging to family S24-7 and genera *Allobaculum*, *Stenotrophomonas*, and *Epulopiscium* were significantly enriched in the malignant group compared to the non-malignant group.

**Conclusion:**

Our results are the first to demonstrate a microbiome signature according to MPE and non-MPE. The role of microbiome in pleural effusion pathogenesis needs further exploration.

## Introduction

Up to 55% of large pleural effusions are malignant pleural effusions (MPE). [1, 2] A majority of these effusions are secondary to metastatic involvement of the pleura from lung cancer and breast cancer. [2–4] However, at a fundamental level, our knowledge for the molecular pathogenesis of initiation and progression of MPE remains poor. There is ongoing research into the development of novel molecular markers that may complement fluid cytology in the diagnosis of malignant pleural effusion (MPE). [1] Conventional pleural fluid cytology on first thoracentesis are positive in only 50% of cases. [2] When the diagnosis is not obtained, repeat thoracentesis and cytological examination merely confers a small additive diagnostic value and more invasive procedures such as thoracoscopic pleural biopsy are required. [3, 4]

Investigations have identified associations between specific microbes and different cancers. The mechanisms by which microbiota modulate carcinogenesis in the lungs, the most adjacent organ to the pleura, is studied in different lung diseases, including lung cancer [5] Pulmonary *Mycobacterium tuberculosis* infection is associated with an increased risk of lung cancer, even among the never-smokers in a meta-analysis of 30 studies, where the pooled relative risk for association between TB and lung cancer was 1.76 (95% CI: 1.49, 2.08). [6] Periodontal disease has also been associated with lung cancer, suggesting an association between the oral microbiome and lung cancer risk. [7] The translocation of microbial pathogens leads to a series of immune responses, which promote carcinogenesis of host cells. [8–11] The estimated trillions of microbes that inhabit the human body establish a beneficial relationship with the host, but it is clear that disruption of this homeostasis (dysbiosis) may result in host disease, including inflammatory disorders and cancers. [12] However, whether there is a specific microbiome associated with MPE formation has not yet been determined.

In this study, we hypothesized that there are distinct differences in the pleural fluid microbiome profile of MPE and non-MPE. In addition, we hypothesized that differences exist among microbiome profile of cancers of different origin. The primary objective of this study was to define discriminating features of the pleural fluid microbiome in malignant and non-malignant pleural effusion. The secondary objective was to compare the microbiome profile associated with metastatic adenocarcinoma of lung cancer origin to metastatic adenocarcinoma of breast cancer origin, in the pleural fluid.

## Methods

### Design and setting

In this exploratory pilot study, from a prospectively enrolled pleural fluid biorepository, pleural fluid specimens of adult patients (> 18 years of age) were included. Written informed consent was obtained from all patients prior to enrollment in the study. The study was approved by the local Institutional Review Board. (IRB # HM14281).

### Inclusion and exclusion criteria

Adult patients (> 18 years of age) with pleural effusion referred for diagnostic and therapeutic thoracentesis were recruited from September 2012- December 2013. Study group included patients with diagnosis of MPE proven on cytology or pleural biopsy. Non-malignant effusion were included as comparators in the control group. Non-malignant effusion was defined as an effusion with negative cytology or pleural biopsy and no evidence of malignancy on 2-year follow up. Patients were excluded if a concomitant second malignancy was present or suspected.

### Data and sample collection

Thoracentesis was performed using sterile standard technique (Details of the aspiration procedure are listed under S1 File). Pleural fluid specimens were collected in sterile fashion and were placed in study test tubes containing 0.1 cc of DNA stabilizing solution. Samples were stored in a secure freezer at—80°C. Once recruitment was completed, specimens were thawed for analysis as described below. Additional recorded parameters were age, sex, race, smoking status, Eastern Cooperative Oncology Group (ECOG) performance status, medical history and chest imaging. All data were stored in a de-identified secure database (REDCap). Specimen processing was performed by laboratory personnel (AS, NG) blinded to the specimens’ origin.

### 16S rRNA gene amplicon library preparation and sequencing

Following DNA extraction using mitochondrial blockers from PNA bio at 2uM per reaction, the V4 region of the 16S rRNA gene (515F-806R) was amplified with region-specific primers that included the Illumina flowcell adapter sequences and a 12-base barcode sequence (Thompson et al., 2017). [13] Each 25μl PCR reaction contained the following mixture: 12μl of MoBio PCR Water (Certified DNA-Free; MoBio, Carlsbad, USA), 10μl of 5-Prime HotMasterMix (1×), 1μl of forward primer (5μM concentration, 200pM final), 1μl of Golay Barcode Tagged Reverse Primer (5μM concentration, 200pM final), and 1μl of template DNA (Thompson et al., 2017). The conditions for PCR were as follows: 94°C for 3min to denature the DNA, with 35 cycles at 94°C for 45s, 50°C for 60s, and 72°C for 90s, with a final extension of 10min at 72°C to ensure complete amplification. [14] Amplicons were quantified using PicoGreen (Invitrogen) assays and a plate reader, followed by clean up using UltraClean® PCR Clean-Up Kit (MoBio, Carlsbad, USA) and then quantification using Qubit readings (Invitrogen, Grand Island, USA). The 16S gene amplified samples were sequenced on an Illumina MiSeq platform at Argonne National Laboratory core sequencing facility according to EMP standard protocols (http://www.earthmicrobiome.org/emp-standard-protocols/its/). [14]

### Internal validity and reagent control

While contamination remains an issue in microbiome studies, we used total of 30 negative controls to identify DNA extraction kit associated contaminants. Ten wells with the sequencing reagents of PCR mix (mentioned above) that were used for processing of experimental specimens were sequenced but without template DNA. The sequencing of the ten reagent control samples generated less than 500 reads per sample (less than 1ng/ul DNA in each well). The dominant sequences in the experimental data had higher mean relative abundances among samples than among non-template controls. This suggests that there was no detectable reagent contamination that could be attributed to the sample processing. The 6 taxa (exact sequence variants (ESVs); <0.1% relative abundance) that were annotated in these ten controls are tabulated in S1 Table. In addition to these, 20 blank controls were also sequenced (only microbial DNA-free water without reagents and template DNA) which failed sequencing due to no detectable DNA, suggesting no attributable contamination from the water used in the PCR mix.

### Data processing and statistical analyses

For 16S rRNA gene based analysis, around 2.5 million paired-end reads generated using Illumina MiSeq were joined using join_paired_ends.py script followed by quality-filtering and demultiplexing using split_libraries_fastq.py script in QIIME 1.9.1. [15] The final set of demultiplexed sequences were then selected for sub-OTU picking using DeBlur pipeline. [16] Taxonomy was assigned by mapping the ESV sequences against the Greengenes reference database (http://greengenes.secondgenome.com/?prefix=downloads/greengenes_database/gg_13_5/) using assign_taxonomy.py script in the QIIME package. In the pipeline, *de novo* chimeras were analyzed, artifacts were removed, and Exact Sequence Variants (ESVs) with under 10 reads were removed using Phyloseq (version-1.26.1) package. [17] The final BIOM file comprised of 2,158 ESVs in 47 samples and average of 7,484 reads per sample was then used for the analyses.

Analyses of patient-level characteristics utilized Pearson χ2 test for categorical data and one-way ANOVA for continuous variables. Non-metric multidimensional scaling (NMDS) plots were employed to reveal beta diversity variations based on weighted and unweighted UniFrac dissimilarity matrices () in Phyloseq (version- 1.26.1) package. [18] Richness, Shannon and Simpson indices were used to estimate alpha diversity and the variation between groups (beta diversity) was statistically tested using paired t-tests and permutational multivariate analysis of variance (PERMANOVA), respectively. Analyses of Composition of Microbiome (ANCOM- version 2) [19] was used to identify differentially abundant bacterial ESVs between the groups at P-value cut-off of 0.05 and Benjamini-Hochberg FDR correction was implemented. Generalized linear models (GLMs) were run in order to test for correlations between selected genera (differentially abundant between groups of interest) and the clinical variables. GLMs were implemented in glm() package (version-1.2.1) using the counts data for the genera using Poisson regression and “log” link without making assumptions about the data distribution. The significance of each correlation for the clinical parameters was evaluated using ANOVA to compare nested models using “chisq” test. For the linear models, standardized beta coefficients were plotted to overcome the bias introduced due to varying scales (units) for the clinical parameters. [20] GLMs were used to quantify the effect (i.e. the direction of correlation and the strength of correlation) of the confounding variables i.e. the clinical variables on microbiome, whereas, ANCOM was used to identify the differentially abundant candidates after adjusting for these confounding variables.

In order to track potential sources/contamination from skin and oral cavity in the pleural microbiome, we employed Bayesian approach using software SourceTracker. [21] We used publicly available data from the National Institutes of Health Human Microbiome Project (HMP) for the sample types buccal mucosa, hard palate, saliva, throat, and tongue dorsum as representatives for the potential contamination from oral cavity and right and left antecubital fossa as representative of skin associated sources. [22] For this analysis, the pleural samples were treated as “sink” which were collapsed into one group i.e. pleural microbiome as well as samples split into two different origin (breast and lung) pleural adenocarcinoma and non-malignant groups for plotting in R script (version- 3.5.1). [23]

## Results

### Population and sampling

Pleural fluid specimens were collected from 38 patients with MPE (study group) and 9 patients with non-MPE (control group). Among the study group, a subgroup of patients with metastatic adenocarcinoma of the pleura were identified (n = 22, adenocarcinoma of lung cancer origin (LA-MPE) = 11, adenocarcinoma of breast cancer origin (BA-MPE) = 11).

The mean (SD) age for the entire cohort was 60 (±8) years and 43% of patients were male. At the time of specimen collection, the 49% of patients were prior smokers, while 40% were never smokers and 11% were current smokers. Most patients had an ECOG performance status of 1–3. Mean (SD) BMI was 25 (0.9). None of the patients had clinical or radiographic evidence of bronchopleural fistula at the time of fluid collection. Patient characteristics by group are shown in Table 1.

**Table 1:** Patients characteristics.

Variables		Malignant (38)	Non-Malignant (9)	
Age (mean) (SD)		61 (1.9)	57 (5.0)	
Sex				
Males		14 (37%)	6 (67%)	
Females		24 (63%)	3 (33%)	
BMI (mean)(SD)		25 (0.9)	26 (1.5)	
Smoking Status				
Former		17 (45%)	6 (67%)	
Never		16 (42%)	3 (33%)	
Current		5 (13%)	0 (0%)	
ECOG				
0		2 (5%)	1 (11%)	
1		9 (24%)	1 (11%)	
2		10 (26%)	5 (56%)	
3		10 (26%)	1 (11%)	
4		4 (11%)	0 (0%)	
Not assessed		3 (8%)	1 (11%)	
Etiology				
	Lung Adeno.	11 (29%)	CHF	4 (44%)
	Lung other	3 (8%)	Renal	2 (22%)
	Breast Adeno	11 (29%)	HH	3 (33%)
	Colon Adeno.	3 (8%)		
	Lymphoma	4 (10%)		
	Other	6 (16%)		

SD: Standard deviation, ECOG: Eastern Cooperative Oncology Group, BMI: Body mass index, Adeno: Adenocarcinoma, HH: Hepatic Hydrothorax, CHF: congestive heart failure, SLE: Systemic Lupus Erythematosus.

### Pleural microbiota composition and comparison

**Fig 1:**
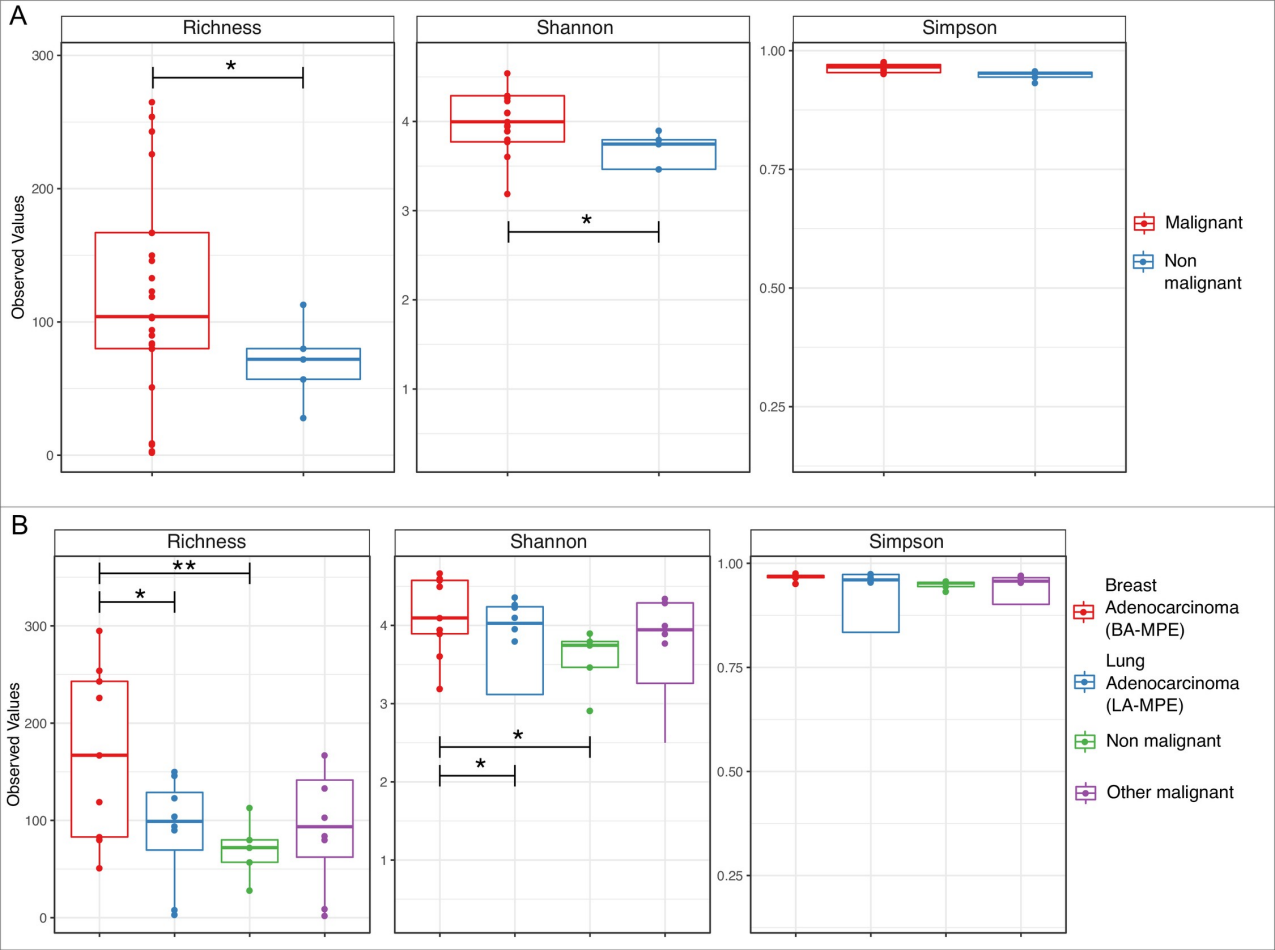
(A) Boxplots showing distribution of Richness, Shannon and Simpson alpha diversity indices between the all malignant effusions and all non-malignant effusion. (B) Boxplots showing distribution of Richness, Shannon and Simpson alpha diversity indices between the breast adenocarcinoma (BA-MPE), lung adenocarcinoma (LA-MPE), non-malignant and rest of the malignant groups. The * and ** represent *p-*values of ≤ 0.05 and *p*-value ≤ 0.01 respectively determined using paired t-test.

There were significant differences in microbial alpha diversity indices between malignant and non-malignant effusions with significantly greater microbial diversity within the malignant group when compared to the non-malignant group based on Richness and Shannon indices. (Fig 1A). Within the MPE group, BA-MPE group had significantly greater microbial diversity compared to the non-malignant group (Fig 1B) based on Shannon and richness indices. Additionally, the BA-MPE group had significantly (p < 0.05; paired t-test) greater alpha diversity when compared with the LA-MPE group based on richness and Shannon indices (Fig 1A). Further, using linear regression and ANOVA test, we identified no statistically significant correlation (*p* = 0.07) between alpha diversity and potentially confounding variables i.e. age, BMI, smoking status, and ECOG performance. Using the NMDS plots, no distinct clustering was noted between the groups based on the beta diversity (for both weighted and unweighted UniFrac distance matrices) (S1 Fig in S1 File). The PERMANOVA testing generated a non-significant p-value for the multi-group comparison based on beta diversity metrics (p>0.05; PERMANOVA).

**Fig 2:**
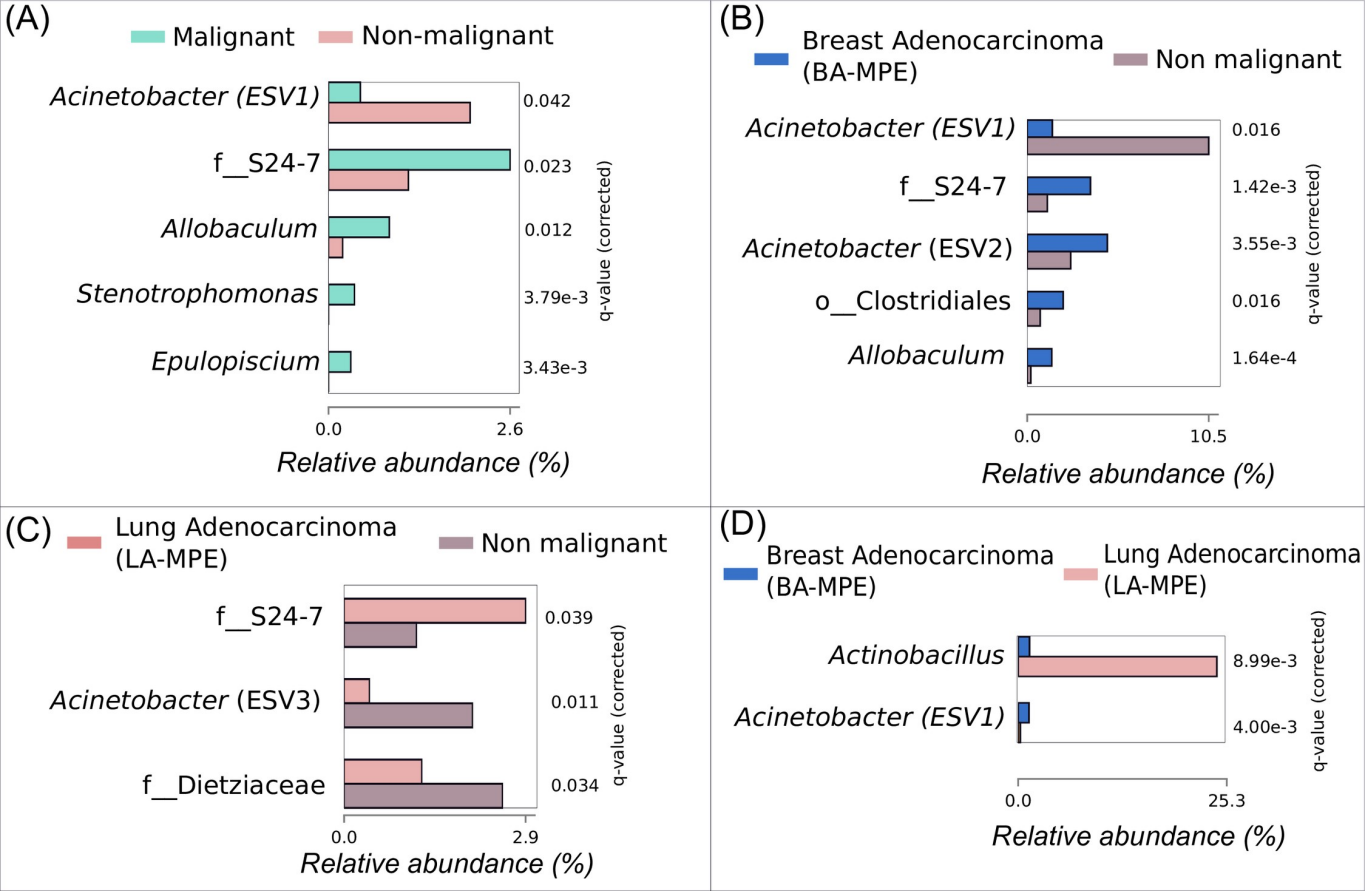
Analyses of Composition of Microbiome (ANCOM) to identify statistically significant Exact Sequence Variants (ESVs) between (A) malignant and non-malignant, (B) lung adenocarcinoma (LA-MPE) and non-malignant group, (C) LA-MPE and BA-MPE, and (D) breast adenocarcinoma (BA-MPE) and non-malignant group. The p-values corrected using Benjamini-Hochberg FDR correction (i.e. q-values) are presented. X-axis shows relative abundance. ANCOM calculations were adjusted for age, BMI, ECOG performance status and smoking status.

Using ANCOM2 and adjusting for age, BMI, smoking status and ECOG performance status, we identified 5 ESVs that could differentiate between the MPE and non-MPE groups. ESV1 annotated to the *Acinetobacter*, and was enriched in the non-malignant group. ESVs belonging to family S24-7 and genera *Allobaculum*, *Stenotrophomonas*, and *Epulopiscium* were significantly enriched in the malignant group compared to the non-malignant group (Fig 2A). We identified 4 ESVs including those annotated to family S24-7, order Clostridiales, genus *Allobaculum* and genus *Acinetobacter* (ESV2) that were significantly enriched in the BA-MPE group compared to non-malignant group, while ESV1 was still significantly enriched in the non-malignant group (Fig 2B). A 3^rd^ *Acinetobacter* ESV (ESV3) was significantly enriched in the non-malignant group compared to the LA-MPE group (Fig 2C). Interestingly, S24-7 ESV enriched in BA-MPE was also significantly enriched in LA-MPE compared to the non-malignant group (Fig 2B and 2C). An ESV annotated as genus *Actinobacillus* was significantly enriched in LA-MPE compared to BA-MPE (Fig 2D).

In order to further elucidate relationships and potential underlying confounders, GLMs were run for the BA-MPE and LA-MPE microbiome (S2 Fig in S1 File). GLMs were used to evaluate any correlation (at 95% CI) between differential abundance patterns of select taxa and variables of age, gender, body mass index (BMI), smoking status and ECOG performance status.

**Fig 3:**
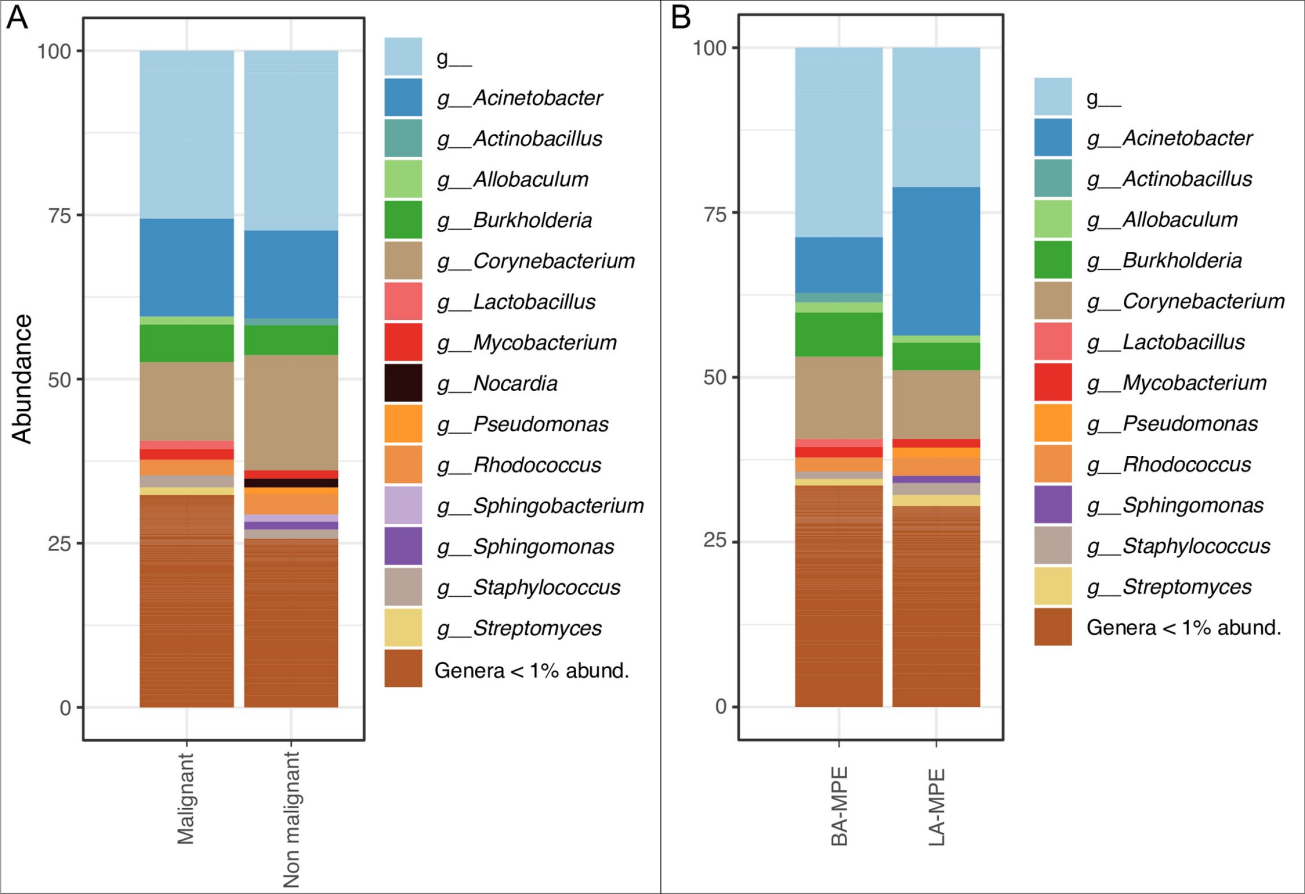
Distribution of all taxa (that are greater than 1% abundant) in the pleural fluid in malignant and non-malignant groups (A) and in BA-MPE and LA-MPE groups.

Furthermore, the pleural fluid taxonomic composition in all MPE, LA-MPE, BA-MPE and non-malignant effusions are displayed. Only taxa greater than 1% relative abundance are plotted in Fig 3.

### Tracking the sources of potential contamination

**Fig 4:**
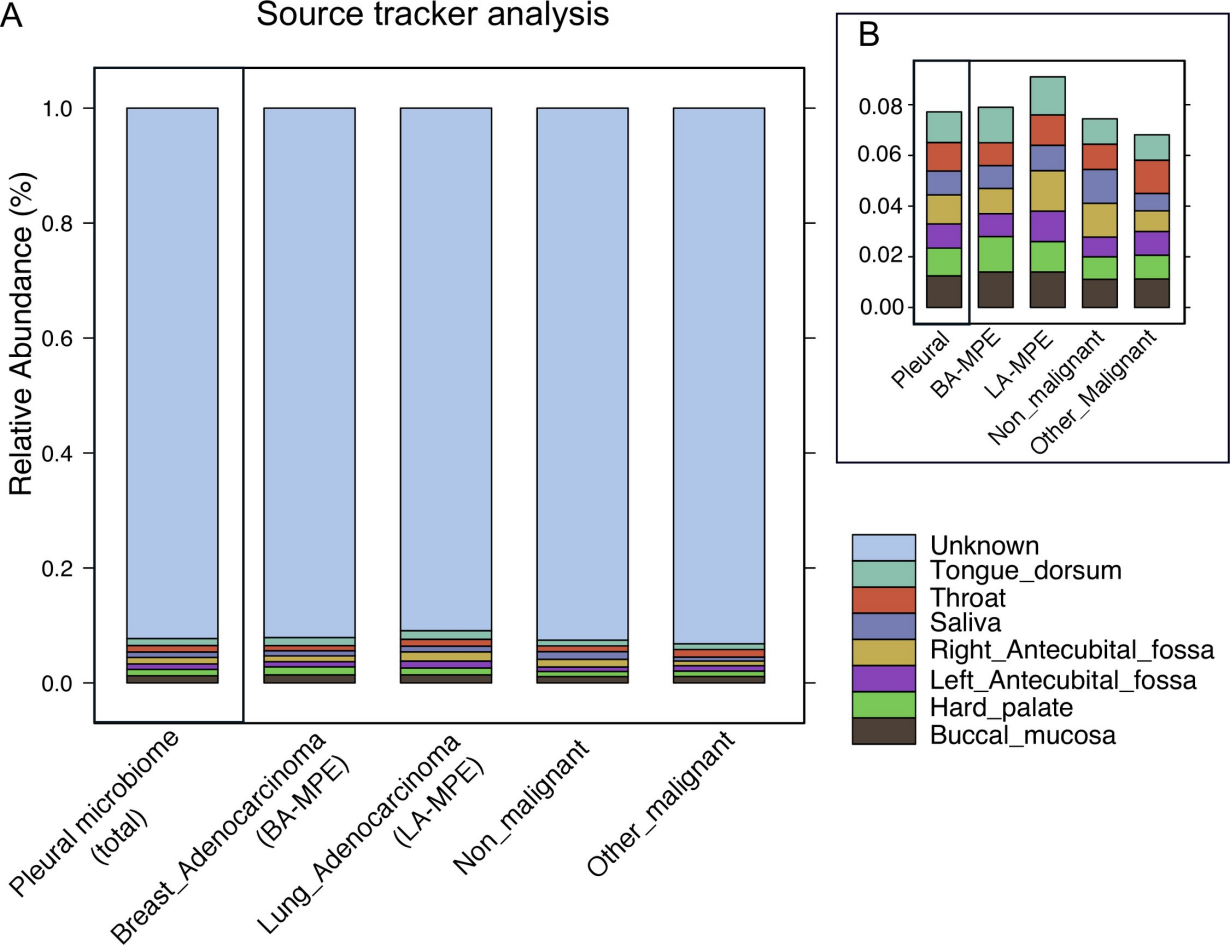
Source tracker analyses results- we used publicly available data from the Human Microbiome Project to identify potential sources (or contamination) of bacterial ESVs enriched in the pleural microbiome. 16S rRNA gene sequencing data from buccal mucosa, hard palate, saliva, throat, and tongue dorsum were used as potential sources for the oral cavity and right and left antecubital fossa as representative of skin associated sources. Here (A) we report the relative proportion distribution of each of the sources in the Pleural microbiome of the total study population (38) as well pleural microbiome data displayed in group-wise fashion. A magnified view of the contribution of each HMP source in each group, is depicted (B).

Source tracking was used to identify the potential origin/sources of the bacterial ESVs enriched in the pleural microbiome. As no prior pleural microbiome reference dataset was available, we used data from the Human Microbiome Project to represent the oral cavity (buccal mucosa, hard palate, saliva, throat, and tongue dorsum) and skin (right and left antecubital fossa) tissue-associated microbiota. These data were used as tissue surrogates for potential sources of contamination from the oral cavity or skin that could occur during pleural resection. These analyses demonstrated a very low contribution (~0.1%) from skin and oral cavity microbiota towards the composition of pleural microbiome, which suggests very low contamination (Fig 4).

## Discussion

These results suggest that there may be a distinct microbial profile in MPE when compared to non-malignant effusions. The variations noted between two different cancer cell origins further suggest that these differences are associated with the tissue subtype driving MPE.

A dysbiotic microbiota may facilitate carcinogenesis through a variety of mechanisms that have been reported in the literature. [13, 24–26] Microbiota are thought to promote carcinogenesis through the release of genotoxins that could result in host-cell DNA damage. [13,24] Bacterial toxins are also thought to promote chronic inflammation triggering damage to host cells. [25] Our preliminary data suggests that not only are there different microbial consortia present in non-malignant and malignant pleural effusion, but importantly there is a distinctive association between different cancer cell origins with a specific microbiome signature. These differences provide valuable biomarkers to improve cell origin prediction, and provide targets to aid in the identification of the contribution of the microbiome to non-malignant and malignant effusion development.

Our analysis in a cohort of pleural effusion specimens from patients with cytology and/or histology proven malignant effusion provided a significantly greater microbial diversity than non-malignant effusions. Additionally, 5 different ESVs were identified that could differentiate between the two groups. Some of these ESVs have been correlated to carcinogenesis in other organs. Wei et al. found an increase in *Ruminococcus obeum* and *Allobaculum*-like bacteria in the feces of rats developing precancerous mucosal lesions. [27] Another study reported that *Stenotrophomonas maltophilia* colonization/infection may be associated with a significant increase in cancer rates over the past two decades. [28] None of the aforementioned studies have shown a relationship between these taxa and pleural malignancy, but their association in other malignancies and models of carcinogenesis is noteworthy.

Cancer of another serous cavity, the peritoneum, and its relationship to microbiome of the peritoneal fluid shows that numerous bacterial taxa are consistently present in patients with pseudomyxoma peritonei. [29] This relation is thought to be secondary to inflammation and translocation of microbial organisms into the peritoneal cavity. We hypothesized that the presence of such inflammatory responses in the pleura and adjacent organisms such as the lung and the diaphragm, could result in microbial translocation in to the pleural space with effects on MPE development.

To assess the overall role of resident airways and oral cavity microbiota, we used oral cavity specimen surrogates from the HMP dataset. Additionally, assuming for possible skin contamination during thoracentesis, we used skin tissue surrogates. Based on our analyses, skin and oral cavity microbiota contribution to pleural microbiome was estimated at approximately 1% only. Other potential sources of direct lung/airway contamination due to pleural injury as a result of alveolar-pleural fistula (APF) and bronchopleural fistula (BPF) were excluded. Hence, majority of the pleural fluid microbiome source identified in our study remains unknown. The results of this study imply the microbiota differs in patients with MPE compared to patients with non-MPE, even after adjusting for age, gender, BMI, smoking status and ECOG performance score.

A small sample size is the main limitation of the present study. This is primarily due to the exploratory nature of microbiome profile investigation in the pleural space which has not been done before. This data aid in power and effect size calculation and the formulation of larger follow-up studies to investigate the role of microbiome as a classifier and its potential association with different pleural disease states. Another limitation is disease heterogeneity in both groups. For this reason, we selected the largest homogeneous groups for additional analyses (LA-MPE and BA-MPE). Analyses were performed within and between groups, to assess both microbial diversity, as well as detect differentiating ESVs. Additionally, multivariable analyses, were performed separately in BA-MPE and LA-MPE groups. Although adjustments were performed to account for numerous potential confounders, other unmeasured confounders could influence the effect estimates and should be included in larger future studies. One of the limitations of microbiome analysis is the possibility of contamination during tissue acquisition. To prevent pleural space contamination during the procedure, all procedures are performed using standard sterile technique. Surface sterilization using standard technique however does not completely prevent the contamination of microbiome. Organisms such as *Myroides*, *Williamsia*, *Brevundimonas*, are known to be on surfaces, water, and commonly found in background. [30, 31]

As an alternate method of source detection and contamination, the HMP data was used. The HMP includes microbial communities from 300 healthy individuals from 5 centers, across several different sites on the human body including the oral cavity and skin. [11] However, the healthy microbial communities in HMP, may not be an accurate representation of oral cavity and skin microbiome in a diseased state. In fact, ESVs belonging to family S24-7 have been associated with airway microbiome in Urethane-induced pulmonary adenocarcinoma in mice [32], While *Stenotrophomonas* and *Acinetobacter* have been associated with bronchiectasis and COPD, respectively. [33, 34]

Finally, the taxonomic composition of the pleural fluid has not been investigated before, hence our lack of knowledge of the pleural space microbiome make-up in a healthy thoracic cavity, prevents a true comparison of MPE with “normal” pleural fluid.

The contribution of microbiome to benign and malignant effusion development should be further investigated. Whether a dysbiotic relationship leads to cancer genesis or, the presence of distinctive microbiome profile, is the result but not the cause of the developing cancer in MPE is unknown. Exploration of the above differences may help expose the underlying molecular mechanisms that drive initiation and progression of malignant pleural effusion and unmask the carcinogenesis process that leads to cancer.

In conclusion, our results link a distinctive association between different cancer cell origins with microbiome and identify different microbial compositions present in non-malignant and malignant pleural effusion. The results of this study should be confirmed in larger prospective studies in homogeneous populations and should include specimens from other potential surfaces and sources of contamination. “Can we manipulate microbial communities to improve clinical outcomes of pleural diseases?” is an intriguing and inevitable question and needs future exploration.

## Supporting information

S1 File (DOCX)

S2 File (ZIP)

S1 Table (XLSX)
