# Supplementary material for: Microbiome profile associated with malignant pleural effusion
Source: PLoS One. 2020 May 8;15(5):e0232181. doi: 10.1371/journal.pone.0232181 (PMC7209204; doi:10.1371/journal.pone.0232181)
Supplement: S1 File — (DOCX) [file pone.0232181.s001.docx]

**Microbiome Profile Associated with Malignant Pleural Effusion**

**Supplementary material:**

Contents:

1. Aspiration procedure
2. Figure 1 with figure legend
3. Figure 2 with figure legend

**Aspiration Procedures**

# All thoracenteses were performed under supervision by an attending physician from the interventional pulmonology team and in a pleural disease negative pressure room. Portable ultrasound was used for examination of the pleural space and puncture site identification. Operator/operators were geared with sterile gown, gloves and wore surgical cap, mask and face shield. All other present personnel who did not directly participate in the procedure wore surgical cap, mask and face shield while in the room per procedure room policy. The identified puncture site and up to at least 10 cm in diameter radius of the surrounding area was prepped twice, each time using ChloraPrep (2% chlorhexidine gluconate in 70% isopropyl alcohol). Following sterile prep, a sterile drape was placed on the patient’s torso, only exposing the puncture site and small portion of the surrounding sterile prepped skin. With the use of 1% lidocaine, local analgesia of the puncture site and deep tissue was achieved. Following this, a soft thoracentesis pleural catheter was inserted and the needle withdrawn using the standard technique (Arrow-Clarke Pleura-Seal Sterile Thoracentesis Kit).  Pleural fluid was removed by manual aspiration.

**Supplementary material - Figure 1: Non-multidimensional scaling (NMDS) plots showing ordination of groups based on beta diversity dissimilarity metrics i.e. weighted and unweighted UniFrac. The p-values were generated using PERMANOVA testing.**

In order to further elucidate relationships and potential underlying confounders, GLMs were run for the BA-MPE and LA-MPE microbiome (Supplementary material, Fig. 2,). GLMs were used to evaluate any correlation (at 95% CI) between differential abundance patterns of select taxa and variables of age, gender, body mass index (BMI), smoking status and ECOG performance status. For the BA-MPE microbiota, we observed significant positive correlation between *Actinomyces* and age. Significant correlations were also noted between BMI and *Corynebacterium, Bifidobacterium pseudolongum, Flavobacterium, Aerococcus, Enterococcus*, and *Oscillospira*. Additionally, *Corynebacterium* and *Flavobacterium* were negatively correlated with ECOG score and *Streptococcus* was negatively correlated with smoking status (Fig. 4). For the LA-MPE microbiome, *Sphingomonas* was positively correlated with BMI while *Ruminococcus* and *Gordonia* were negatively correlated with ECOG score. *Streptomyces, Prevotella* and *Kingella* showed significant negative association with smoking status (Supplementary material, Fig. S2).

**Supplementary material - Figure 2: Generalized linear models (GLMs) to estimate association between the differentially abundant ESVs (generated from ANCOM) and the variables of interest i.e. age, BMI, ECOG score, and smoker status. The *, **, *** stand for p-value ≤ 0.05 and p-value ≤ 0.01, and p-value ≤ 0.001. The p-values were corrected using Benjamini-Hochberg FDR correction.**
